# CNNAI: A Convolution Neural Network-Based Latent Fingerprint Matching Using the Combination of Nearest Neighbor Arrangement Indexing

**DOI:** 10.3389/frobt.2020.00113

**Published:** 2020-09-17

**Authors:** Uttam U. Deshpande, V. S. Malemath, Shivanand M. Patil, Sushma V. Chaugule

**Affiliations:** ^1^Department of Electronics and Communication Engineering, KLS Gogte Institute of Technology, Belagavi, India; ^2^Department of Computer Science and Engineering, KLE Dr. M. S. Sheshgiri College of Engineering and Technology, Belagavi, India

**Keywords:** convolution neural network (CNN), CNNAI, global features, nearest neighbor, indexing, feature vector, FVC2004, NIST SD27

## Abstract

Automatic fingerprint identification systems (AFIS) make use of global fingerprint information like ridge flow, ridge frequency, and delta or core points for fingerprint alignment, before performing matching. In latent fingerprints, the ridges will be smudged and delta or core points may not be available. It becomes difficult to pre-align fingerprints with such partial fingerprint information. Further, global features are not robust against fingerprint deformations; rotation, scale, and fingerprint matching using global features pose more challenges. We have developed a local minutia-based convolution neural network (CNN) matching model called “Combination of Nearest Neighbor Arrangement Indexing (CNNAI).” This model makes use of a set of “n” local nearest minutiae neighbor features and generates rotation-scale invariant feature vectors. Our proposed system doesn't depend upon any fingerprint alignment information. In large fingerprint databases, it becomes very difficult to query every fingerprint against every other fingerprint in the database. To address this issue, we make use of hash indexing to reduce the number of retrievals. We have used a residual learning-based CNN model to enhance and extract the minutiae features. Matching was done on FVC2004 and NIST SD27 latent fingerprint databases against 640 and 3,758 gallery fingerprint images, respectively. We obtained a Rank-1 identification rate of 80% for FVC2004 fingerprints and 84.5% for NIST SD27 latent fingerprint databases. The experimental results show improvement in the Rank-1 identification rate compared to the state-of-art algorithms, and the results reveal that the system is robust against rotation and scale.

## Introduction

**Figure 1:**
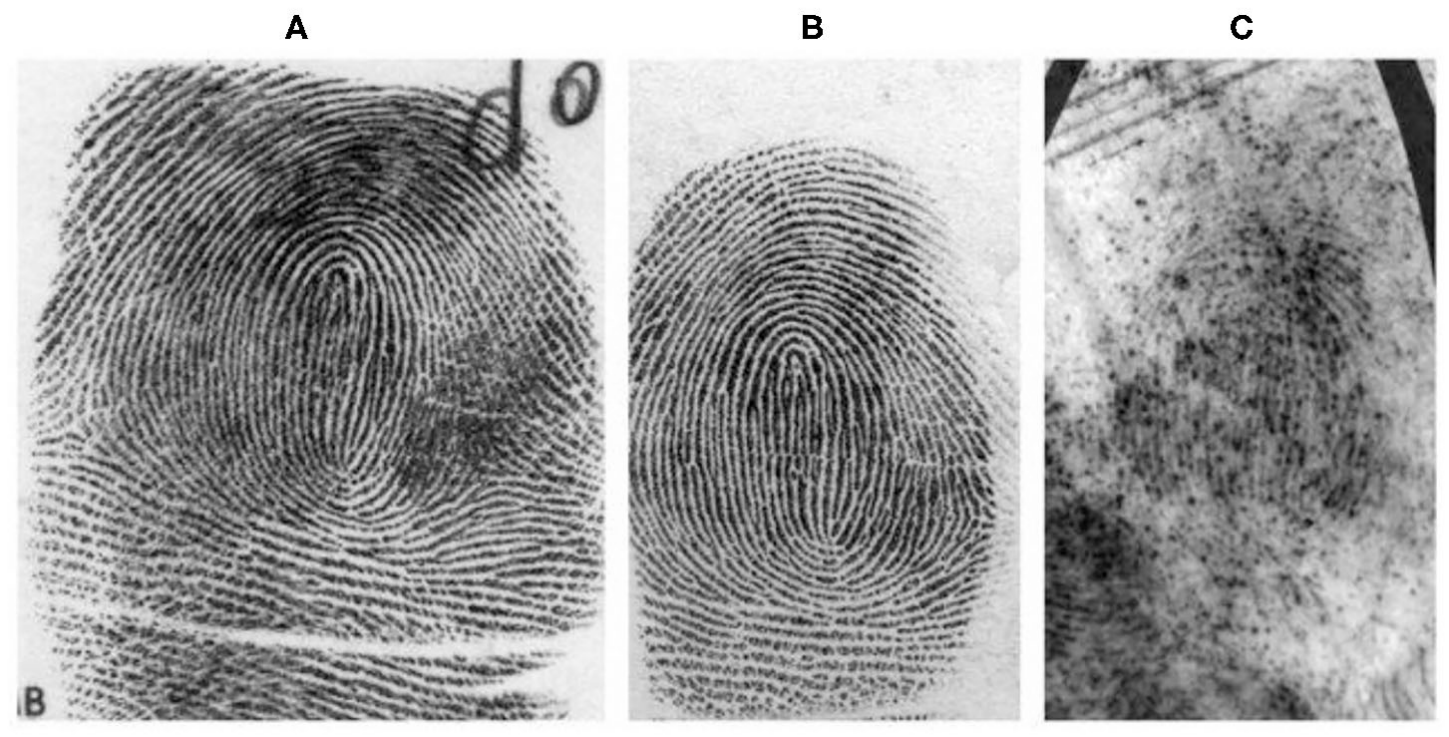
Types of fingerprint images (Garris and Mccabe, 2000): **(A)** Rolled, **(B)** Plain obtained from scan method, and **(C)** Latent fingerprints of the same finger obtained from the crime scene.

A fingerprint is one of the more popularly used biometrics used in-person identification (Lee and Gaensslen, 2001). This is because fingerprints are easy to collect, examine, and classify. No two persons have been found with the same fingerprints and are found to be unique. Fingerprint characteristics never change throughout the age of a person. Fingerprints are more unique than DNA. Although identical twins share the same DNA, they can't have the same fingerprints. As seen in Figure 1, fingerprint images are classified into three categories. They are rolled, plain, and latent fingerprints. To capture the complete ridge information, a finger is rolled from one side to another side. The fingerprint obtained using this method is called as “rolled” fingerprint, whereas to obtain the plain fingerprint impressions, the fingerprint is pressed down against the flat plane surface. Compared to rolled fingerprints, the plain fingerprint impressions cover a small fingerprint area and introduce fewer distortions. Rolled or plain impressions are obtained by capturing the inked impression from paper or captured from scanning devices. On the other hand, latent fingerprints are the impressions obtained from article types on the surface of different objects. These impressions are unintentionally left over the surface. Latent fingerprints play a very important role in the criminal investigation.

Manual observations of a fingerprint are prone to inconsistency and can lead to errors (Ulery et al., 2011). With the increasing size of fingerprint databases, AFIS is becoming more popular for fingerprint identification in law enforcement applications (Maltoni et al., 2009). The use of AFIS by law enforcement agencies has significantly improved the matching accuracy of fingerprint identification. AFIS has been successfully tested for matching fingerprints on forensic applications as well as many civilian and commercial applications (Lee and Gaensslen, 2001; Maltoni et al., 2009). Fingerprint matching utilizes one of the following search strategies (Jain and Feng, 2011): (i) ten-print search and (ii) latent search. In a ten-print search, the fingerprint recognition involves searching for 10 fingerprints of a person against a known fingerprint database. Hence, this method is more suitable for civil applications; whereas a latent search involves identifying a person using the fingerprint obtained from a crime scene against the fingerprint database of unknown persons. Latent fingerprints are the unintentionally left fingerprints on the objects and are directly not visible to human eyes. Thus, the latent search strategy is more suitable for law enforcement agencies for criminal investigation.

Unlike rolled fingerprints, latent fingerprints suffer from non-linear distortions with unspecified fingerprint orientations due to uneven pressure (Zhu et al., 2017) on the surface. Due to poor quality of latent, very few minutiae points will be available for matching, and most of the time, singular points such as core and delta will not be present in the obtained latent fingerprints. Fingerprint matching using poor latent poses more challenges compared to rolled fingerprint matching. Most of the rolled fingerprint matching methods proposed by researchers rely on the fingerprint features mentioned above. Latent fingerprint matching utilizes global features (Jain et al., 2008; Jain and Feng, 2011; Yoon et al., 2011) such as ridge information and ridge frequency along with local features such as singular points for fingerprint alignment. The latent fingerprint matching methods are explained in detail next.

### Minutiae Based Latent Fingerprint Matching Systems

Latent fingerprint matching based on the fingerprint features are classified as follows:

➢ **Correlation-based matching:** In this approach, two fingerprints are superimposed to calculate their similarity between corresponding pixels at different fingerprint alignments.
➢ **Minutiae-based matching:** This feature is the most reliable and popularly used fingerprint feature. Minutiae are extracted from a fingerprint image and are put on a 2D plane. The matching is carried out by comparing the minutiae arrangement between the fingerprint in the gallery database against the query fingerprint.
➢ **Non-Minutiae feature-based matching:** Minutiae extraction is challenging in most latent fingerprint images. Global features like ridge flow are used to identify the reliability of minutiae. This is method is not reliable and not popularly used.

Minutiae-based matching is more reliable compared to the other two methods (Jain and Feng, 2011). Methods proposed by most of the researchers rely on manually marked fingerprint features (Yoon et al., 2010; Feng et al., 2013). The latent to rolled/plain matching algorithm (Jain and Feng, 2011) proposed depends on the manually marked fingerprint features like minutiae, core points, and delta. The algorithm was tested for the NIST SD27 database, and Rank-1 identification accuracy of 74% was reported. Fingerprint matching using an orientation field and quality map (Jain et al., 2008) was developed for matching. ARank-1 identification accuracy of 79.5% was achieved for NIST SD27 fingerprints. A latent fingerprint enhancement algorithm using a manually marked region of interest (ROI) and singular points (Yoon et al., 2011) was proposed, and it produced a Rank-1 identification accuracy of about 38% with a background database of 27 k fingerprints.

A Minutia Cylinder-Codes (MCC) (Cappelli et al., 2011) based Hough Transform (Paulino et al., 2013) was proposed for latent fingerprint identification. MCC is considered to be one of the state-of-art indexing techniques, which performs matching at the local level and fingerprint alignment through the Hough Transform. To improve the matching accuracy and non-linear distortions, clustering (Angel Medina-Pérez et al., 2016) based on minutiae cylinder codes (MCC), M triplets, and neighboring minutiae-based descriptors (NMD) was proposed. The algorithm merges overlapping minutiae clusters to matching minutiae, and the descriptor rotation was restricted to π/4. The highest rank-1 accuracy of 82.9% was reported by the NMD clustering algorithm.

Deep learning is applied to latent fingerprints for image enhancement (Tang et al., 2017) and feature extraction (Nguyen et al., 2018; Deshpande and Malemath, in press). Rank-1 identification of 35% was reported (Tang et al., 2017) for 40 k large background fingerprints of the NIST SD27 database. A minutia descriptor-based convolution neural network (CNN) model called “ConvNets” (Cao and Jain, 2019) produced a Rank-1 identification accuracy of 51.2% for NIST SD27 databases.

A patch-based latent fingerprint matching using deep neural networks without handcrafted features was developed (Ezeobiejesi and Bhanu, 2018). The system follows the patch representation and patch similarity method for matching. A Rank-1 identification rate of 81.35% was reported for this method. The patch-based system occupies large memory space to incorporate patches obtained from different angles from a sample fingerprint. Other approaches rely on manually marked fingerprint features for fingerprint alignment before matching.

To summarize, manual fingerprint matching is time-consuming and may lead to errors. AFIS has shown significant improvement in matching results compared to manual matching. The existing AFIS developed for latent fingerprint identification requires prior ridge information for fingerprint alignment before performing matching. With incomplete ridge information and distorted ridges, it becomes difficult to accurately align fingerprints to perform matching. To overcome these problems, an automatic latent fingerprint identification system called “Convolution Neural Network-Based Combination of Nearest Neighbor Arrangement Indexing (CNNAI)” has been proposed. This proposed system can identify a person with few minutiae points, and it does not depend upon global features to perform matching. We use simple CNN representation to classify the fingerprint match based on the geometrical arrangement. The geometrical minutiae arrangements are obtained from annotated latent fingerprints (Feng et al., 2013). Instead of rotating image patches (Ezeobiejesi and Bhanu, 2018), we propose rotation and scale-invariant local minutiae arrangement vectors to assist matching. The algorithm is explained in the next section. The paper is organized as follows: Section Combination of Nearest Neighbor Arrangement Indexing (CNNAI) algorithm introduces the Combination of Nearest Neighbor Arrangement Indexing (CNNAI) algorithm. Section CNNAI model proposes a CNN-based CNNAI model. Section Training and Testing the CNNAI model deals with training and testing the CNNAI model. Section Results and Discussion describes the experiments carried out on plain and latent fingerprint databases, and section concludes the work.

## Combination of the Nearest Neighbor Arrangement Indexing (CNNAI) Algorithm

For latent fingerprint matching, we use the nearest combination of minutiae points around a central minutia. We obtain the discriminative invariants based on the minutiae structures and store them on the hash-table for matching. To make the matcher robust against scale, rotation, and missing minutiae, we define the triangular minutiae structure. The minutiae arrangement vector and its CNN implementation are discussed next.

### Local Minutiae Arrangement Vector

**Figure 2:**
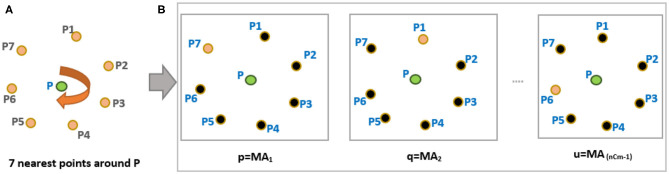
**(A)** Arrangements of “n” nearest points in a clockwise direction **(B)** Possible combinations of 6 minutiae points (*m* = 6) from the nearest 7 minutiae points (*n* = 7).

**Figure 3:**
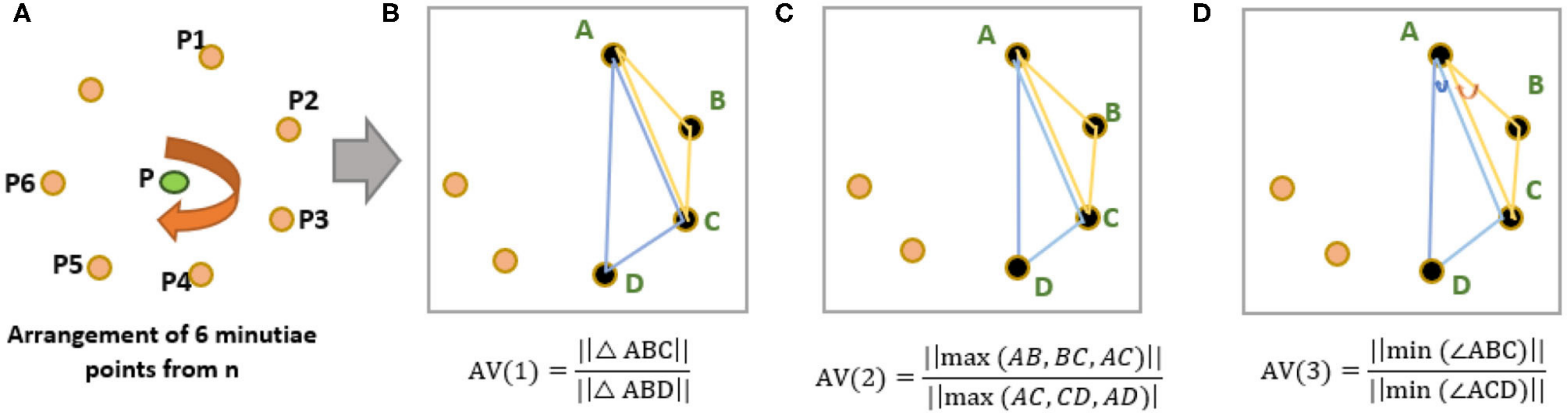
**(A)** Arrangements of “*m*” nearest minutiae points from n points (*n* = 7, *m* = 6). **(B)** 1st invariant AV(1). **(C)** 2nd invariant AV(2). **(D)** 3rd invariant AV(3).

**Figure 4:**
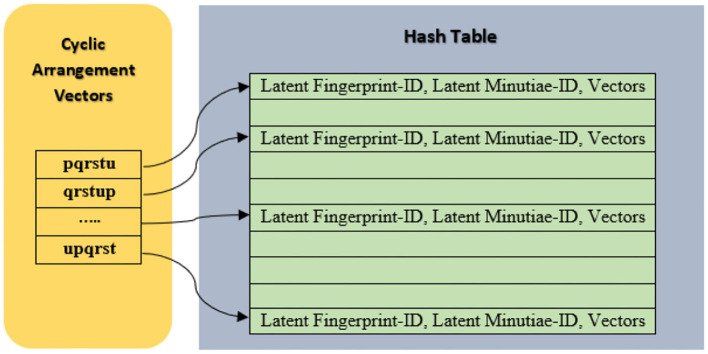
Hash-Table construction for arrangement vectors.

We define a fixed-length minutiae descriptor from its distinctive minutiae neighborhood arrangement. This helps us to match two fingerprints without the knowledge of global alignment information. We start with a reference minutia (P) to calculate arrangement as shown in Figure 2A. The steps are as follows:

- Calculate 7 nearest neighbors (*n* = 7) around a reference minutia “P” from Figure 2A.
- Choose six minutiae points (*m* = 6) from “n” points such that m<n. For example, six points P1, P2, P3, P4, P5, and P6 are chosen around P (see Figure 3A). With *n* = 7 and *m* = 6, the total possible number of minutiae arrangements (MA) is equal to “nCm-1” combinations, and they are MA_1_, MA_2_, MA_3_, …, MA_6_. For convenience, we use p, q, r, s, t, u notations to indicate all possible “nCm-1” minutiae arrangements (see Figure 2B).
- Select a minutiae arrangement (from Figure 2B) and calculate the weighted average (WAvg) values of all the arrangement vectors. Initially, we chose “p” minutiae arrangement and select four points (*k* = 4) A,B,C,D to calculate three arrangement vectors AV(1), AV(2), and AV(3) (see Figures 3B–D) as follows:
  (1) $$\begin{array}{l} AV\left(1\right)=Ratio\;of\;area\;of\;triangles\\ \;=\frac{||\Delta ABC||}{||\Delta \text{ABD}||} \end{array}$$
  (2) $$\begin{array}{l} AV\left(2\right)=Ratio\;of\;largest\;sides\;of\;triangle\\ \;=\frac{||\text{max}\left(\text{A}\text{B},\;\text{B}\text{C},\;\text{A}\text{C}\right)||}{||\text{m}\text{a}\text{x}\left(\text{A}\text{C},\;\text{C}\text{D},\;\text{A}\text{D}\right)|} \end{array}$$
  (3) $$\begin{array}{l} AV\left(3\right)=Ratio\;of\;minimun\;triangle\;angle\\ \;=\frac{||\text{m}i\text{n}\left(∠\text{ABC}\right)||}{||\text{m}i\text{n}\left(∠\text{ACD}\right)||} \end{array}$$
  Triangular features are chosen to calculate arrangement vectors to make the system robust against the scaling of a fingerprint image. Finally, the WAvg value of AV (1), AV (2), and AV (3) is calculated for this given minutia arrangement.
- A, B, C, and D points are rotated in a clockwise direction above “p” minutiae arrangement to calculate WAvg from remaining arrangement vectors (using step iii). With *m* = 6 and *k* = 4, the total number of arrangement vectors for a given minutiae arrangements is calculated as,
- $\text{mC}4=\left(\begin{array}{c} \text{m}\\ 4 \end{array}\right)=15$.
- After all the arrangement vectors are calculated, the Hash-index for this minutiae arrangement is calculated using equation 4.
  (4) $$\begin{array}{l} HTindex=\left(\sum_{\text{i}=0}^{\left(\text{mC}4\right)-1}\text{WAvg }\left(\text{i}\right).{\text{q}\text{n}}^{\text{i}}\right){\text{moHT}}_{\text{size}} \end{array}$$
  Where qn is Quantization value and qn = 13, HT_size_ is Hash-Table size and HT_size_ = 2^32^-1
- Repeat step iii–vi for the remaining minutiae arrangements (q,r,s,t,u) for a reference minutia “P” chosen in step i.
- Cyclically arrange the obtained arrangement vectors for “m” points as pqrstu, qrstup, rstupq, ……, upqrst in the Hash-Table as shown in Figure 4. This step is used to make the system robust against fingerprint rotation.
- Repeat steps i–viii for the remaining minutiae points in the same fingerprint.

This procedure is followed while registering latent fingerprints in the database as well as while testing query fingerprints. The arrangement vectors of query fingerprints are compared against the stored vectors. The voting method is used to increase the vote count for matching minutiae belonging to a particular fingerprint. The arrangement vector count of different fingerprints is sorted in decrement order, and the count with the highest voting is chosen as Rank-1 retrieved fingerprint.

## CNNAI Model

**Figure 5:**
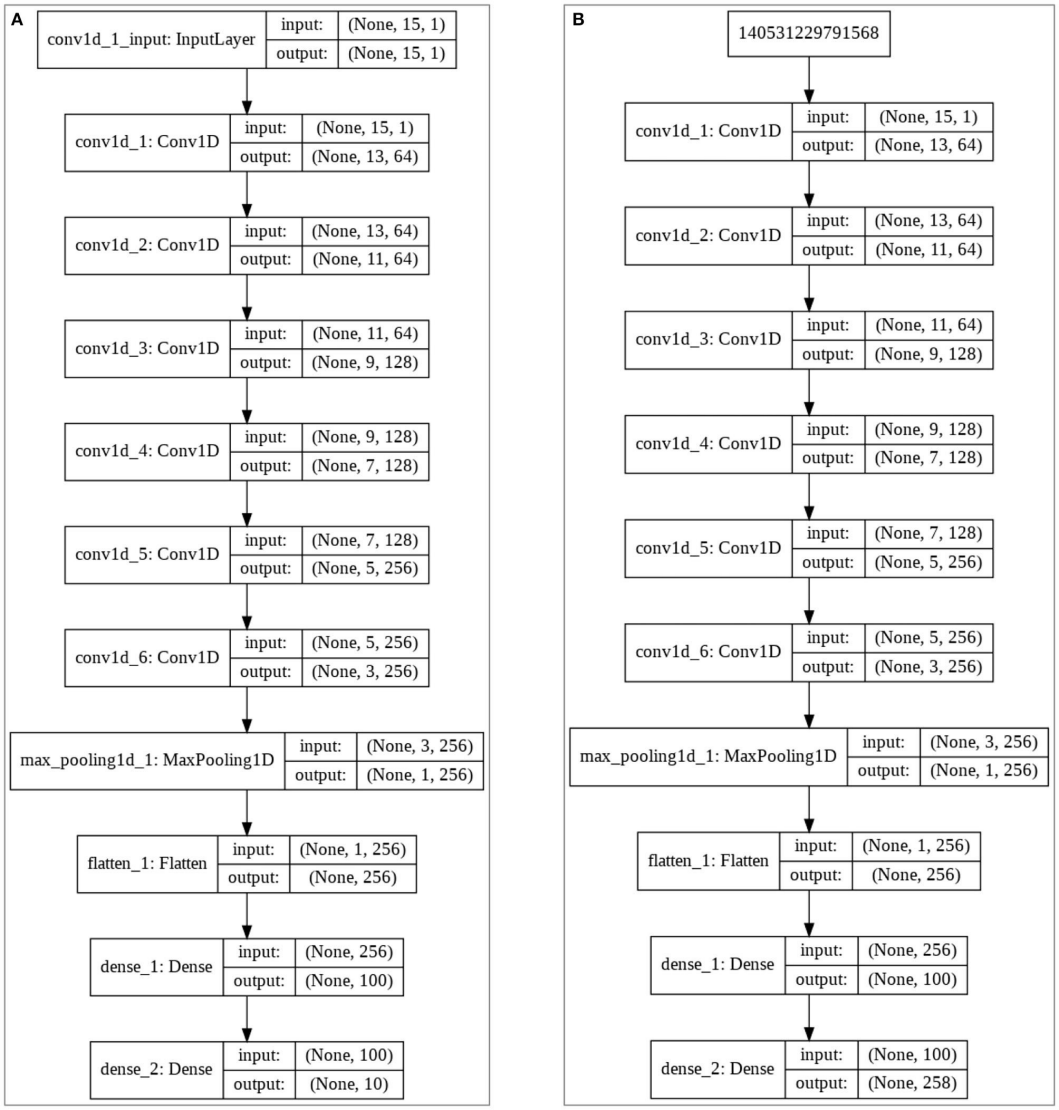
CNNAI matching model **(A)** FVC2004 **(B)** NIST SD27.

We develop a CNN-based matching model for the CNNAI algorithm discussed in section Combination of Nearest Neighbor Arrangement Indexing (CNNAI) algorithm. The proposed matching model employs neural network techniques for classifying a query latent fingerprint from a class of a given set of pre-trained classes depending upon the arrangement vectors. One-dimensional convolutional layer is used in designing the matching model. CNN makes use of simple computational units and is connected by weighted links via which the activation values are transmitted. Computational units calculate these new activation values from the past received connections. For matching a latent query fingerprint, this fingerprint is fed into the network as an activation to some of the input units. CNN is connected to a web of other network units via a connection that results as an activation to the output unit. Finally, this results in the matching output. Figures 5A,B explain the proposed matching model for FVC2004 (Maio et al., 2004) and NIST SD27 (Garris and Mccabe, 2000) fingerprints.

During the fingerprint training phase, the arrangement vectors for all NIST SD27, NIST SD4, and FVC2004 images are generated by the indexing method. These arrangement vectors for the query fingerprint are obtained, and these vectors form the input for the model, whereas the corresponding image for these feature vectors forms the output. Input, output, and weights of the model are adjusted to classify the image. As seen in Figure 5, “Softmax” is used as the activation function in the last layer of the matching model.

### Softmax

**Figure 6:**
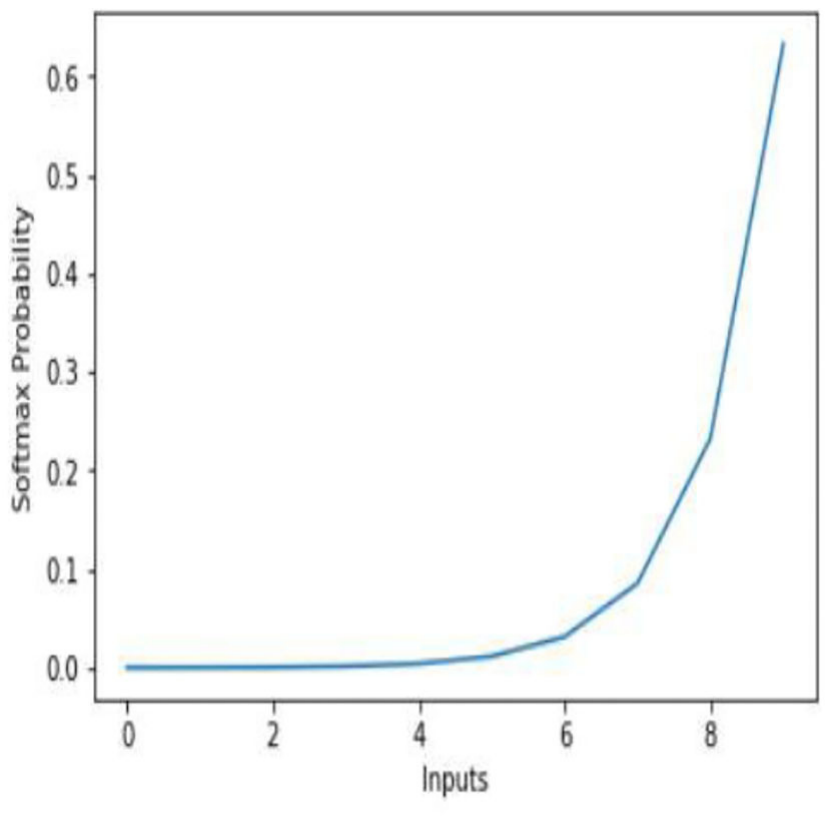
Softmax activation function.

Softmax is used to handle the multi-class problem. It is used to compute the probability distribution of input to different “n” classes. This is a non-linear function and is used to decide the class to which the input belongs to. The Softmax classifies by outputting value between 0 to 1. Here, “0” indicates no feature of an image is matching to the relevant class, and “1” indicates all features of an image are matching the particular class to which image belongs to (see Figure 6). In our model, convolution layers are used for recognizing the patterns within the data. The feature vectors are passed through the multilayer convolutional network followed by the “Relu” activation function. The connection weights are determined during the training of the model with the help of fingerprint label and arrangement vectors. As discussed earlier, every fingerprint is characterized by multiple arrangement vectors obtained from the combination of nearest neighbor arrangement indexing. Each arrangement vector is provided as an input to the neural network model. The Softmax function outputs each of these arrangement vectors with the probability of values for different labels. All the arrangement vectors are matched, and the label that has obtained the highest match is outputted as the fingerprint match. The model parameters for the proposed CNNAI model are listed in Table 1.

**Table 1:** Model parameters for the CNNAI.

**Name**	**Type**	**Dimension**	**Filter**	**Stride**
Input	input	64 × 1	–	–
Conv	Convolution	–	2, 64 2, 128 2, 256	–
ReLu	ReLu	–	–	–
MaxPool	Max Pooling	2	–	1
Softmax	Softmax	–	–	–
Output	Match Probability			

*Epochs: 300, Batch size: 100, Learning rate: 0.001*.

## Training and Testing the CNNAI Model

### Gallery Dataset

To improve the quality of images, we have used the CNN-based model called “MINU-EXTRACTNET” Deshpande and Malemath (in press) to enhance, extract the minutiae location, and obtain the orientation of query fingerprints. MINU-EXTRACTNET is trained with a total of 8,000 images including plain (3,200 images) and augmented images from the FVC 2002 dataset (Maio et al., 2002). To perform matching, we created a gallery database of 640 fingerprints obtained from FVC2004 and FVC2002 databases. Similarly, for NIST SD27, the gallery database of 3,758 fingerprints is obtained from NIST SD27 and SD4 databases. For these gallery databases, the minutiae are extracted, and indexes are generated using the CNNAI algorithm. The algorithm produces arrangement vectors and the document id's as the output for these gallery fingerprints. To prevent the overfitting of the model, separate models are generated for FVC2004 and NIST SD27 separately.

### Training, and Testing the Model

The training of the CNNAI model was done using Categorical Cross-Entropy with a batch size of 100 and varying learning rates for the model. The primary objective of choosing categorical cross-entropy is the classification of a single label. The input weight of all hidden layers is the output weights for the respective preceding layers.

We adjusted the learning rate to minimize the cross-entropy error and it is defined as:

(5) $$\begin{array}{l} L\left(y,\;\hat{y}\right)=-\sum_{j=0}^{M}\sum_{i=0}^{N}\left(yij*log\;\left(\hat{y}ij\right)\right) \end{array}$$

Where y is the observed value and y is the predicted expected value. Categorical cross-entropy is used to compare the distribution of the predictions. It predicts the activations in the output layer, one for each class with the true distribution. The probability of the true class is set to 1 and is set to 0 for the other classes.

**Figure 7:**
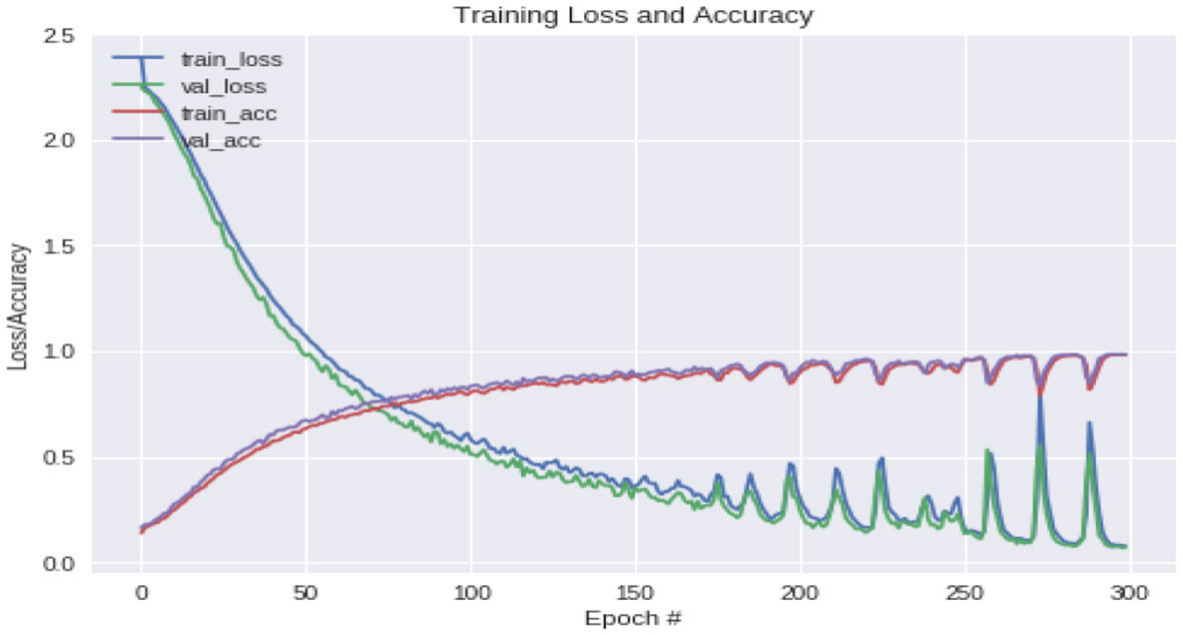
Training loss and accuracy plot.

The true class is represented as a one-hot encoded vector. As the model's output is closer to that vector, the losses will be lower. We use categorical cross-entropy together with the softmax activation function. We used 23,000 minutia samples to train, 5,000 minutia samples for validation and 3,300 minutia samples to test the network. There was no overlap between the training, validation, and test datasets. We obtained 97.33, 93.75, and 88.9% of training, validation, and testing accuracy, respectively. We used 70% of the database for training, 20% for validation, and 10% of the database for testing the results. Figure 7 indicates the loss and accuracy learned on the NIST SD27 database during training by the CNNAI.

## Results and Discussion

Experiments were conducted on FVC2004 and annotated NIST SD27 (Feng et al., 2013) query fingerprint images. FVC2004 is a challenging database with a latent-fingerprint-type quality of images. FVC2004 contains 80 fingerprints from 10 persons (class) with each person registering 8 fingerprints. Hence it contains a total of 10 fingerprint classes. NIST SD27 is a criminal fingerprint database and contains 258 fingerprint images obtained from 258 persons. It forms a total class of 258 fingerprint images with 88 Good, 85 Bad, and 85 Ugly images.

The quality of latent fingerprints is affected by non-linear distortions and suffer from variations in fingerprint shape. Before extracting minutiae, we used image enhancement steps on FVC2004 and NIST SD27 query fingerprint databases to overcome these problems. The enhancement process contains image normalization, local orientation estimation, local frequency estimation, region mask estimation, and Gabor filtering steps. Later, minutiae features were extracted using MINU-EXTRACTNET Deshpande and Malemath (in press), and the indexes are generated using the CNNAI algorithm. The algorithm produces arrangement vectors and the document IDs as the output for these query fingerprints. All query fingerprints were tested with the CNNAI model to obtain matching results.

Because of the poor quality of latent fingerprints, the classifiers face a major challenge while classifying the fingerprint class. After the minutiae features are extracted, softmax loss tries to push the features away for the fingerprints belonging to a different class (inter-class). To deal with the intraclass problem, we use center loss (Wen et al., 2016), which pulls the features belonging to the same class closer after extracting minutiae.

**Figure 8:**
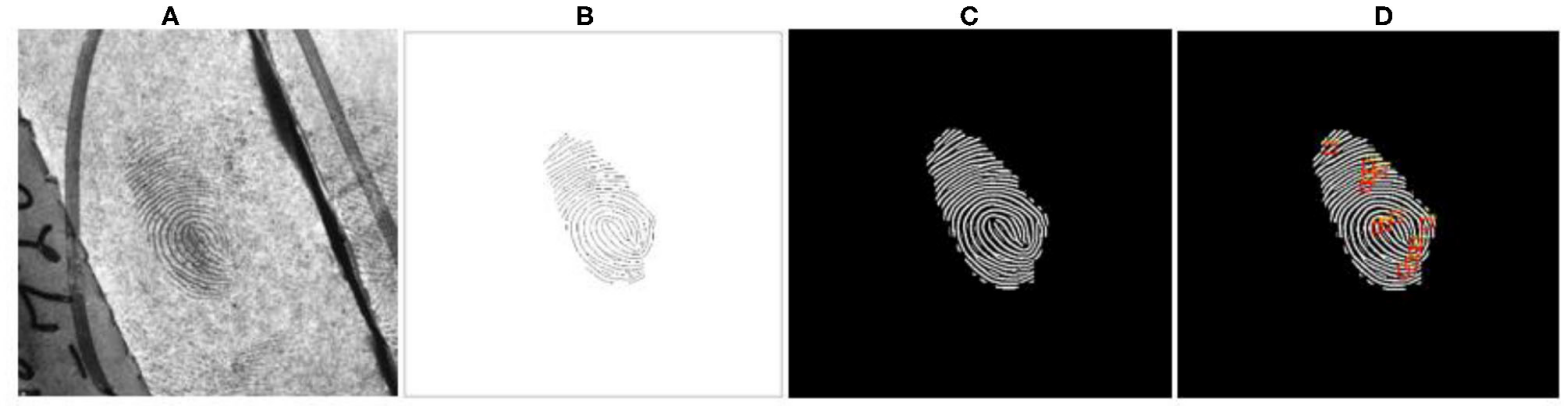
Minutiae extraction of NIST SD27 query fingerprints **(A)** Original fingerprint. **(B)** Annotated skeleton image. **(C)** Preprocessed and Enhanced image. **(D)** Minutiae extracted by MINU-EXTRACTNET (Deshpande and Malemath, in press).

Figure 8 shows the minutiae extraction steps followed in our proposed work for NIST SD27 query fingerprints. Similarly, we use MinutiaeNet to extract minutiae points for FVC2004 query fingerprints.

**Figure 9:**
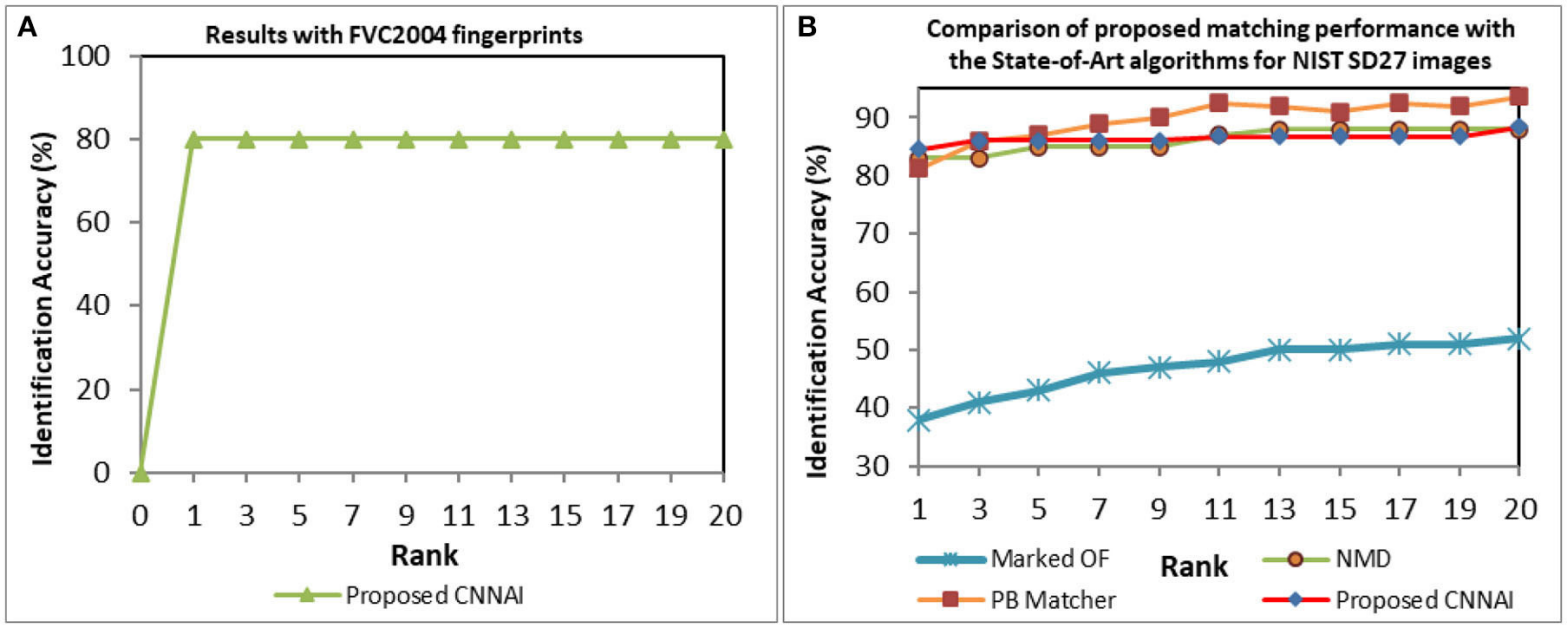
CMC curves indicating fingerprint matching results **(A)** FVC2004 database. **(B)** NIST SD27 database.

We performed matching experiments by testing 640 and 3,758 background gallery images of FVC2004 and NIST SD27 fingerprints against query fingerprints. To test the robustness of the proposed system, all 80 FVC2004 and 258 NIST SD27 query fingerprints are randomly rotated and scaled before performing matching. We obtained a Rank-1 identification accuracy of 80% as shown in Figure 9A. We compared the performance of our model for the NIST SD27 database with state-of-art algorithms (Feng et al., 2013; Angel Medina-Pérez et al., 2016; Ezeobiejesi and Bhanu, 2018) (see Figure 9B). Our proposed CNNAI model produced a Rank-1 identification accuracy of 84.5% over 81.5% from patch-based (Ezeobiejesi and Bhanu, 2018) and 83% from NMD (Angel Medina-Pérez et al., 2016) algorithms.

**Figure 10:**
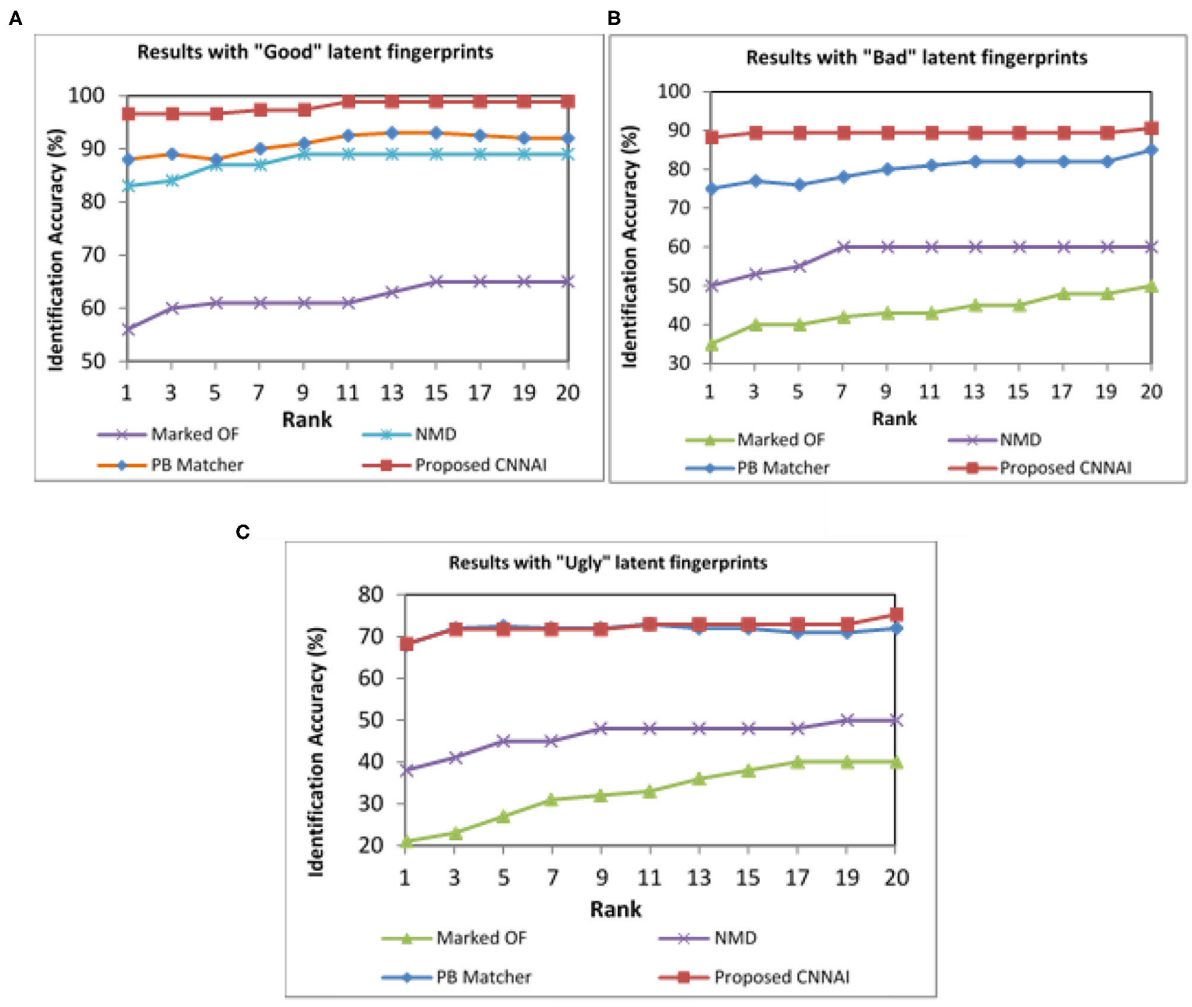
CMC curves indicating different qualities of NIST SD27 fingerprint matching results. **(A)** Good quality. **(B)** Bad quality. **(C)** Ugly quality images.

NIST SD27 Good, Bad, and Ugly images were tested separately, and the performance was evaluated. Our proposed model achieved a Rank-1 identification accuracy of 96.59% over 88% from patch-based and 83% from NMD algorithms for Good quality images (see Figure 10A). Our proposed model achieved a Rank-1 identification accuracy of 88.24% over 75% from patch-based and 50% from NMD algorithms for Bad-quality images (see Figure 10B). Similarly, our proposed model achieved a Rank-1 identification accuracy of 68.24% over 68% from patch-based and 38% from NMD algorithms for Ugly-quality images (see Figure 10C).

### Confusion-Matrix

The confusion matrix shown in Table 2 is used to measure the performance of the two-class problem. For fingerprint matching problems, a correct match is treated as a “positive class,” and an incorrect match is treated as a “negative class.” The right diagonal elements classify the True Positive (TP) and True Negative (TN) instances, whereas left diagonal elements classify False Positive (FP) and False Negative (FN) instances incorrectly. TP is used to obtain correct positive fingerprint prediction, and FP is used to measure incorrect positive fingerprint prediction, whereas TN provides correct negative fingerprint prediction and FN provides incorrect negative prediction.

**Table 2:** Confusion matrix indicating four possible results (A) FVC2004 (B) NIST SD27.

**PREDICTED**					
**Observed**		**A**		**B**	
	**Class**	**Positive**	**Negative**	**Positive**	**Negative**
	Positive	64	8	228	1
	Negative	16	72	30	257

To obtain a confusion matrix for our proposed model gallery, fingerprint datasets with two different classes are formed. For FVC2004 query fingerprints, a gallery fingerprint dataset with 160 instances is created by choosing 80 fingerprints from FVC2004 and 80 fingerprints from FVC2002.

For NIST SD27 query fingerprints, a gallery fingerprint dataset of 516 instances is created by choosing 258 latent fingerprints from the NIST SD27 dataset and 258 fingerprints from the FVC2002 dataset. The fingerprint matching for FVC2004 and NIST SD27 query fingerprints is done, and the four instances, namely, TP, TN, FP, and FN, are obtained. The results are tabulated in Table 2.

For testing, fingerprints from these two classes are randomly chosen. The confusion matrix in Table 2 shows the actual and predicted classification. For a given class of FVC2004, the total number of true positives is 64, and false positives, 16. For the other class, the total number of false positives is 8, and false negatives, 72. Similarly, for a given class of NIST SD27, the total number of true positives is 228, and false positives, 30. For the other class, the total number of false positives is 1, and false negatives, 257. Table 3 indicates various measures calculated from the instances obtained from the confusion matrix.

**Table 3:** Other measures derived from the confusion matrix.

**Parameters**	**FVC2004%**	**NIST SD27%**
Error rate	15.00	6.01
Accuracy (ACC)	85.00	93.99
Sensitivity (True Positive Rate—TPR)	88.89	99.56
False negative rate (FNR)	11.11	0.44
Specificity (True negative rate—TNR)	81.82	89.55
False positive rate (FPR)	18.18	10.45
Precision (Positive Predictive Value)	80.00	79.44
Recall	88.89	99.56

### Receiver Operating Characteristics (ROC)

**Figure 11:**
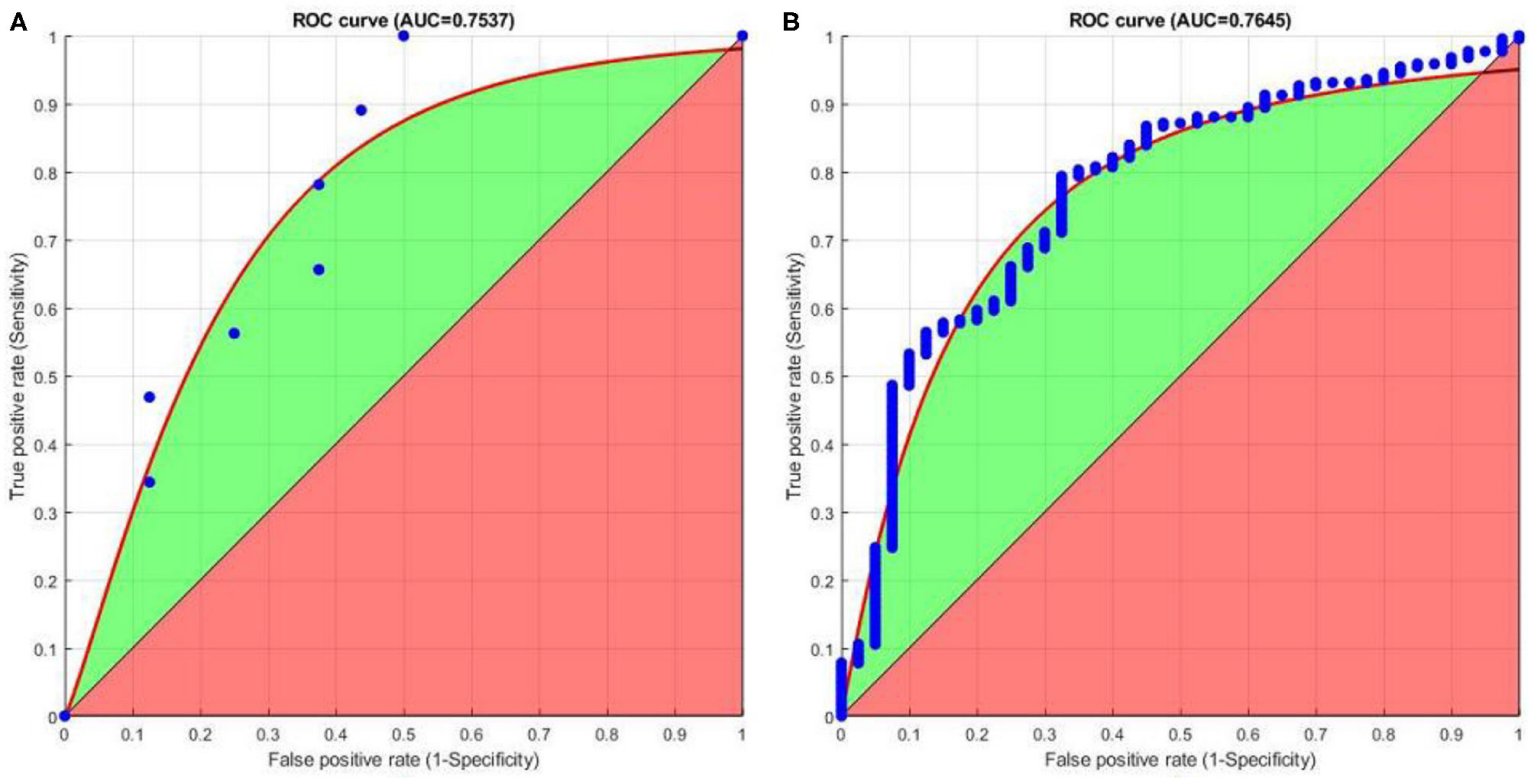
ROC curve for **(A)** FVC2004 dataset. **(B)** NIST SD27 dataset.

Finally, we plot receiver operating characteristics (ROC) based on the obtained matching results. The ROC graphs provide a useful technique for organizing classifiers and helps in visualizing their performance. The area under ROC curve (AUC) gives the probability of a classifier that will rank a randomly chosen positive instance higher than a randomly chosen negative one. Figure 11 shows that good AUC values are obtained for both FVC2004 and NIST SD27 datasets. Overall, the proposed CNNAI produces good positive matching results.

## Conclusions and Future Work

Latent fingerprint matching is a challenging step in latent fingerprint identification. Latent fingerprints suffer from non-linear distortions and the quality of latent fingerprints is poor. The fingerprint does not contain full ridge information, and hence, it is not available in its complete form. This results in fingerprints ending up with very few or improper minutia information. To perform matching, most of the existing AFIS depends upon global features like core or delta points for fingerprint pre-alignment. Latent fingerprints may not always contain these global features, and it becomes difficult to perform matching. We have proposed a CNN-based automatic latent fingerprint matching system called “CNNAI” that works on local minutia features. The CNNAI algorithm works on the existing nearest neighbor minutiae arrangement structure and generates rotation and scale-invariant minutiae arrangement vectors based on these arrangements. This eliminates the need for fingerprint pre-alignment required to perform matching. Hash-indexes based on these feature vectors are generated and stored inside the hash table. Fingerprints receive votes based on the number of votes and are sorted in descending order. Top 20 retrieved fingerprints produce Rank-20 identification results. Further, we developed a CNN-based matching model for the proposed CNNAI algorithm. Our proposed model learns the minutiae representation based on its arrangement and predicts the fingerprint matching. We tested the performance of the algorithms on FVC2004 and NIST SD27 fingerprint databases and obtained a Rank-1 identification rate of 80 and 84.5%, respectively. During experimentation, all query fingerprints were randomly rotated and scaled. The experimental results show that the proposed model produced improved results compared to the reported state-of-art algorithms, and the system is robust against rotation and scale. ROC curves indicate that the CNNAI performs a good matching operation for both databases. The advantage of the CNNAI model is that it can be easily integrated with any minutiae extraction system that generates minutiae in the form of x, y, and theta.

**Figure 12:**
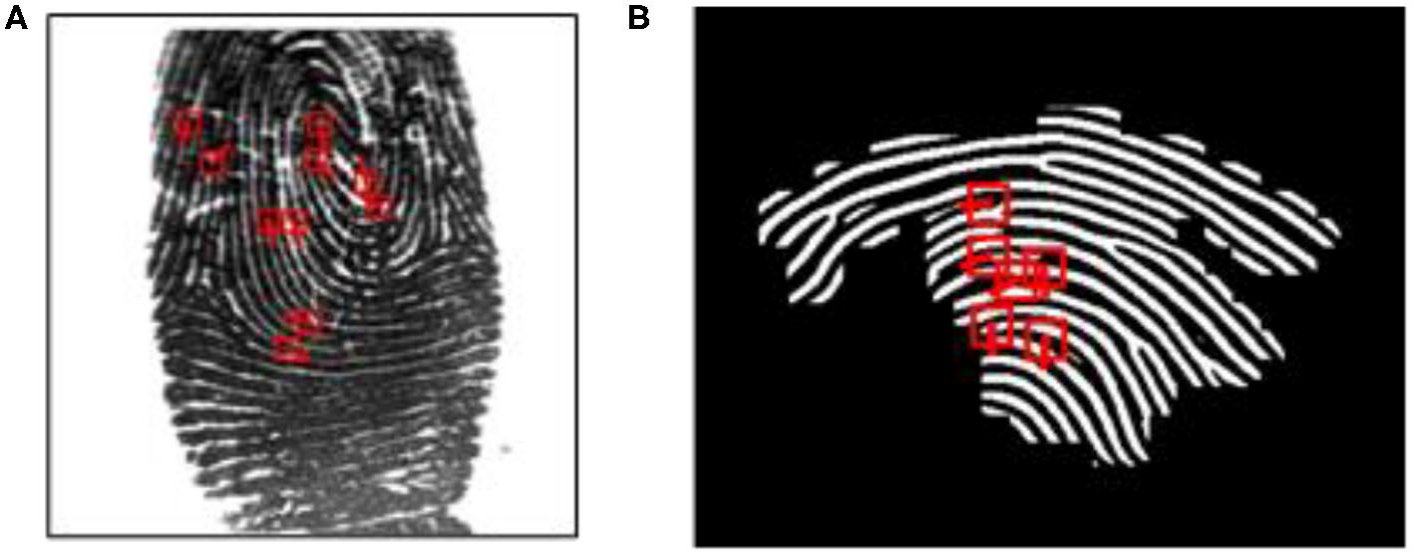
An unidentified latent fingerprint of NIST SD27 by CNNAI. **(A)** Original. **(B)** Enhanced.

As discussed earlier, latent fingerprint matching is a challenging step in latent fingerprint identification. This is mainly because the quality of the latent fingerprints is poor and the fingerprint itself may not be always available in its complete form (see Figure 12A). Figure 12B shows an enhanced and annotated fingerprint of Figure 12A. Our proposed CNNAI failed to identify the latent fingerprint shown in Figure 12B. The main reason is that there are a small number of minutiae points available in the fingerprint and the deep neural-network-based robust minutiae extractor MINU-EXTRACTNET Deshpande and Malemath (in press) failed to extract all minutiae points from the fingerprint. For CNNAI to perform a fingerprint identification, at least eight minutiae points should be available to generate an arrangement vector before matching. It becomes crucial for a minutiae extractor to extract all possible minutiae points in the small or partial fingerprint area. An improved minutiae extractor can overcome this problem. To improve matching accuracy, the model can be trained with a large fingerprint database. Furthermore, an end-to-end deep-neural-network model can be developed for complete fingerprint minutiae extraction and matching.

## Data Availability Statement

Publicly available datasets were analyzed in this study. This data can be found here: http://bias.csr.unibo.it/fvc2004/download.asp.

## Author Contributions

UD and VM were involved in surveying the existing work. We explored the possibility of indexing using Nearest Neighbor arrangement for fingerprints. We were involved in analyzing and designing the CNN based model to implement this minutiae based arrangement indexing. We conducted experiments on NIST SD27 and FVC2004 fingerprints. We analyzed the obtained results with the other state-of-art algorithms. We further carried out testing of algorithms with FVC2002 and NIST SD4 databases to obtain the confusion matrix and carried out further analysis. All authors contributed to the article and approved the submitted version.

## Conflict of Interest

The authors declare that the research was conducted in the absence of any commercial or financial relationships that could be construed as a potential conflict of interest.
